# Physiological responses and early hormonal signals associated with growth-defense trade-offs in tomato seedlings under wind-sand stress

**DOI:** 10.3389/fpls.2026.1778781

**Published:** 2026-04-07

**Authors:** Haobo Liu, Jinyue Yang, Shuangji Cai, Ruiyuan Yu, Haiyang Guo, Qinming Sun

**Affiliations:** Agricultural College, Shihezi University, Shihezi, China

**Keywords:** growth-defense trade-off, hormones, mechanical injury, tomato, wind-sand stress

## Abstract

**Introduction:**

Tomato is one of the most widely cultivated vegetable crops worldwide, with China as the leading producer. Xinjiang is a major tomato-producing region; however, it is frequently exposed to persistent wind and sand conditions that severely threaten seedling establishment in early spring.

**Method:**

This study investigated the effects of combined Wind-sand stress on tomato seedling growth using controlled wind-tunnel experiments. Seedlings were subjected to varying intensities of wind alone (3, 5, 7, 9, 11, and 13 m/s) and sand-laden wind (5, 7, 9, 11, and 13 m/s). Changes in physiological parameters, morphology, and endogenous hormone levels (IAA, GA, Zeatin, ABA, JA, and SA) were analyzed to assess stress responses and the growth–defense balance.

**Result:**

The results showed that (1) Both wind alone and sand-laden wind inhibited photosynthesis, with sand-laden wind exerting a more pronounced effect. At 13 m/s, the net photosynthetic rate (Pn) decreased by 71% under wind alone and by 98% under sand-laden conditions compared with the control. (2) Sand-laden wind caused more severe membrane damage, accompanied by higher antioxidant enzyme activities and greater accumulation of osmolytes. (3) High-intensity wind–sand stress resulted in mechanical injury, leading to systemic physiological imbalance and loss of cellular homeostasis. For example, exposure to sand-laden wind at 11 m/s damaged 89% of leaves and stomata, caused complete cuticle exfoliation, and induced abnormal physiological responses, including increased intercellular CO_2_ concentration and reduced enzymatic activity. (4) Sand-laden wind significantly increased stress-related hormones, with jasmonic acid, abscisic acid, and salicylic acid rising by 6,126.3%, 205%, and 302.6%, respectively, while significantly suppressing the synthesis of growth-promoting hormones such as auxin, gibberellins, and zeatin. These pronounced hormonal changes indicate that wind–sand stress triggers a defense-oriented physiological reprogramming at the expense of growth, providing strong hormonal evidence for a growth–defense trade-off in tomato seedlings.

**Meaning:**

This study elucidates the key physiological and hormonal Responses associated with combined wind-sand stress and provide a theoretical basis for stress-resilient cultivation of processing tomatoes and for the development of field-level wind damage mitigation strategies in wind–sand-prone regions.

## 1 Introduction

Wind is an important ecological factor in natural ecosystems that influences plant growth, reproduction, distribution, survival, and ultimately, evolutionary processes (Nobel et al., 1981; Anten et al., 2010). When strong winds blow over dry, loose sand, they generate sand-laden winds, also known as wind-blown sand. Wind–sand activity is a common phenomenon in arid and semi-arid regions (Luo et al., 2026). Wind alone and sand-laden wind have a significant impact on plant growth and development, resulting from multifaceted stress induced by mechanical stimulation, wind erosion, and sand burial, as well as the disruption of key physiological processes, including water regulation and gas exchange (Onoda and Anten, 2014; Liu et al., 2023). Seedlings are particularly vulnerable due to their slender stems, underdeveloped phloem and vascular bundles, and delicate leaf epidermal tissues. Exposure to wind-sand stress at the seedling stage can cause physical damage, resulting in cell-sap exudation, which severely inhibits seedling growth and may even lead to mortality (Zhao et al., 2015; Wang et al., 2025b).

The effects of wind on plant seedlings vary depending on the plant species and wind speed, duration, and frequency (de Langre, 2019). Wind can directly affect plant morphology and physiological function through mechanical stress, leading to water deficit, stem lodging, branch breakage, or even uprooting (Telewski, 1995). Wind can also trigger physiological cascades by altering the growth microenvironment of the plant in terms of humidity, temperature, and gas concentrations (Lin et al., 2022). While sand-laden wind and wind alone stress exhibit similar effects, sand-laden wind causes substantially greater damage to plant seedlings, primarily due to the mechanical abrasion and physiological disturbances induced by solid particles transported in high-velocity air currents (Armbrust and Retta, 2000). The mechanical damage inflicted by sand-laden wind results from various factors, including erosion, sand burial, abrasion, penetration, and breakage (Gardiner et al., 2016). This effect is further associated with the obstruction of light to photosynthetic tissues and the inherent physicochemical characteristics of the airborne particles (Li et al., 2023). Concurrently, physiological harm primarily manifests as a reduction in photosynthetic activity, increased transpiration, and damage to membrane systems. In response, plants activate antioxidant defenses, accumulate osmoregulatory compounds, and exhibit alterations in biomass allocation. In severe cases, these effects can lead to plant death (Mittler and Blumwald, 2015; Zhou et al., 2021).

Xinjiang (China) is one of the largest tomato-growing regions in the world. Together with California (USA) and Italy, it ranks among the leading producers of processed tomatoes (Cammarano et al., 2022). However, this region experiences intense sand and wind-related environmental hazards, especially in oasis agricultural zones along the southern desert margin, characterized by strong winds, abundant sand, and arid conditions, and frequently experiences sandstorms in spring (Pi et al., 2017). While farmland shelterbelts can reduce wind speed and stabilize sand sources, their protective effects are significantly reduced during winter and spring when the trees have lost their leaves (Brandle et al., 2004). During the annual spring plowing period, surface soils become exposed, loose, and dry due to freeze-thaw cycles and agricultural tillage. Under these conditions, frequent strong winds generate intense sand-laden winds, directly threatening agricultural production (Ge et al., 2025; Zhang et al., 2025). Wind and sand disasters can result in seeds being scattered or excessively buried, preventing successful germination. Even when the seedlings do emerge, persistent sand-laden wind exposure causes mechanical damage, tissue dehydration, and destruction of the photosynthetic organs, frequently causing seedling mortality and significant economic impacts (Tao et al., 2022). Wind and sand disasters now constitute a significant natural disaster, restricting agricultural production and development in Xinjiang.

Recent studies have indicated that gentle winds contribute to healthy seedling growth (Yang et al., 2025), but excessive wind speeds cause both mechanical damage and alterations in hormone levels (Liu et al., 2022; Wang et al., 2025a). Furthermore, studies have shown that both wind alone and sand-laden wind can damage the ultrastructure of leaves (Reis et al., 2018; Shi et al., 2023), highlighting the complexity of wind and sand-laden wind stress effects on plants, encompassing multi-level regulatory changes in morphology, physiology, and hormonal responses. When plants experience stress, they often redistribute resources via hormonal pathways to provide a dynamic balance between growth and defense (Züst and Agrawal, 2017). At the core of the growth–defense trade-off lies hormone crosstalk, wherein defense-related hormones such as JA and SA typically antagonize growth-promoting hormones like IAA and GA (Huot et al., 2014; Gan et al., 2025). This hormonal interplay enables plants to prioritize resource allocation toward either growth or defense in response to environmental cues (Nguyen et al., 2016). Consistent with this framework, Cunha et al. demonstrated that mechanical injury in tomato activates JA signaling, suppressing growth while enhancing defense traits (Cunha et al., 2023). However, the unique composite stress imposed by wind and sand encompasses physical impacts, mechanical forces, and drying effects and poses a unique and formidable challenge. The mechanisms underlying its ability to trigger distinct hormonal signaling crosstalk and drive resource reallocation remain unexplored, and its downstream pathways and regulatory processes are equally unclear.

Current research on the effects of wind-sand stress on plants has focused primarily on desert vegetation or tall tree species, with relatively limited investigation of crops (Gao et al., 2023), and studies on the effects of wind-sand stress on horticultural crops are even scarcer. Moreover, most studies are limited to describing morphological and physiological indicators. Investigation of microstructural damage to crops and their associated hormonal response mechanisms under wind-sand stress still requires in-depth exploration. Moreover, the mechanisms underlying the growth–defense trade-off in tomato seedlings exposed to this stress are largely unclear. Clarification of the effects of wind alone and sand-laden wind on tomato seedlings and their physiological responses has significant practical and economic value for tomato cultivation and production in regions frequently affected by sand-laden wind disasters. Therefore, this study hypothesized that the presence of sand particles renders sand-laden wind more damaging to tomato seedlings than wind alone, and that seedlings respond to this combined stress by hormonally mediated reprogramming of the growth-defense balance. To verify the hypothesis, this study used wind tunnel experiments to simulate stress induced by different intensities of wind alone and sand-laden wind on tomato plants. The effects of these two stressors on the physiological functions of tomato seedlings were investigated, with particular emphasis on assessing damage at both macro- and microscopic levels, as well as evaluating alterations in endogenous hormone levels under high-intensity stress. Furthermore, the study elucidated the regulatory mechanisms underlying mechanical damage and the plant growth-defense balance from the perspective of interactions among multiple hormones, aiming to provide a reference for tomato cultivation and the mitigation of wind- and sand-induced damage in sand-laden wind-prone regions.

## 2 Materials and methods

### 2.1 Experimental materials and conditions

To exclude the influence of natural wind, the investigation was conducted in a Multi-Span Solar Greenhouse at Shihezi University during 2024 and 2025. The greenhouse conditions were controlled, with a temperature range of 20–30 °C and relative humidity of 30–50%, while the ambient CO_2_ concentration was maintained at 390–410 μmol/mol, exposure to natural light. The tomato variety used was ‘Shi Fan 33’, a widely cultivated processing tomato cultivar in Xinjiang. The seeds were disinfected and sown in hole trays, and the seedlings were raised in an artificial climate incubator. During seedling cultivation, the diurnal temperature was maintained at 25/18 °C, the photoperiod at 14/10 h (light/dark), and the light intensity at 300 μmol/m^2^/s. All seedlings received uniform water and fertilizer, by daily irrigation to maintain soil moisture at approximately 50%. The experiment began when the seedlings reached the six-leaf stage, with one central bud, at an average height of ~20 cm and a stem diameter of ~0.4 cm. Healthy seedlings of uniform growth were selected and transplanted individually into 10×10×10 cm pots, with three pots forming one replicate. The sandy soil used in the experiment was collected *in situ* from tomato-growing regions in southern Xinjiang. After air-drying, the soil was passed through a 20-mesh sieve to remove large debris. The soil was primarily sandy loam, with a particle size of ≤1 mm. Preliminary tests indicated that the sand-entrainment wind speed for the dry test soil was approximately 4.8 m/s.

### 2.2 Wind tunnel

**Figure 1:**
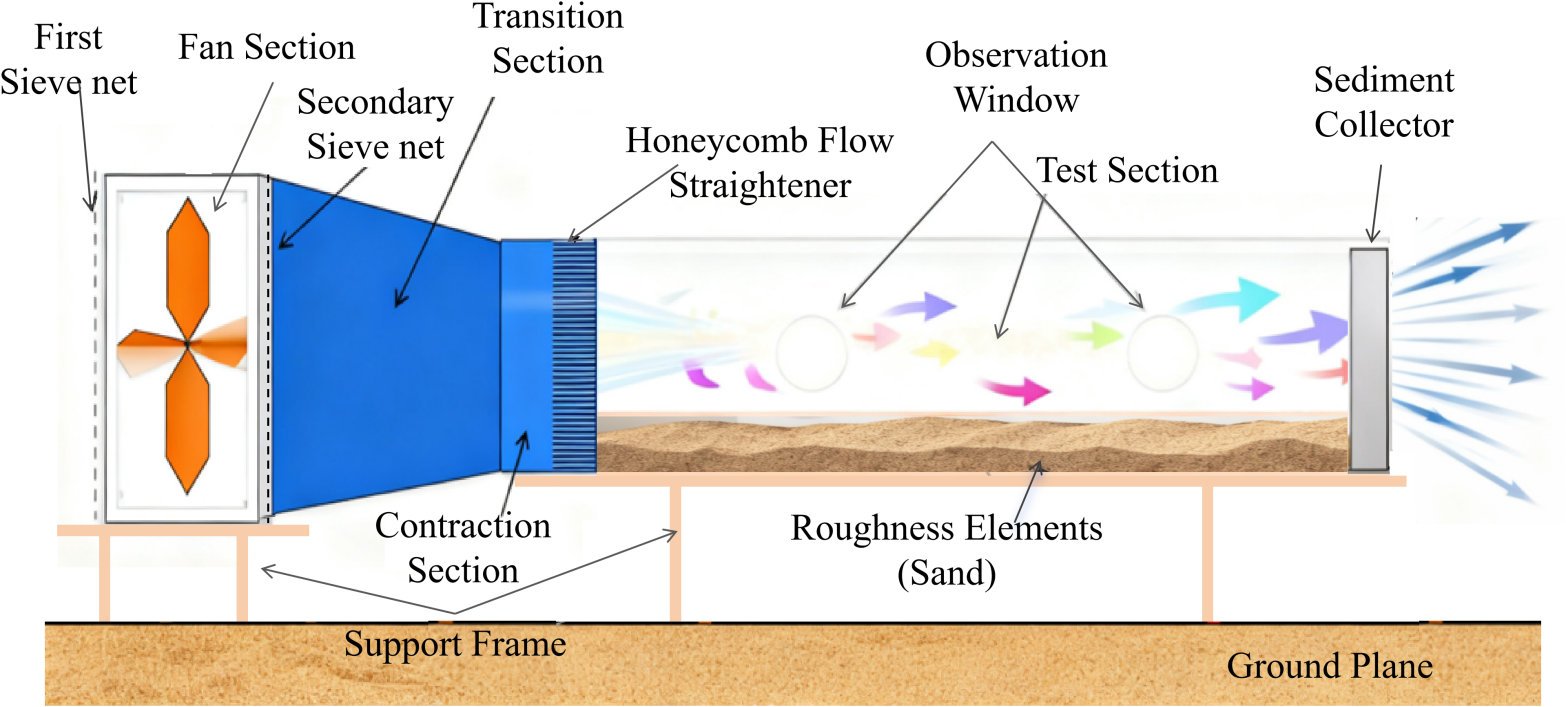
Schematic diagram of the custom-built wind tunnel used in the experiment.

The homemade portable DC wind tunnel is illustrated in **Figure 1**. The total length of the wind tunnel was 3 m, with a core test section measuring 1.6 m (length) × 0.5 m (height) × 0.3 m (width), and was constructed from transparent acrylic panels. The fan operated at 1450 r/min, with a maximum flow rate of 18,700 m³/h. The wind speed in the test section was continuously adjustable from 1 to 13 m/s using a 0–60 Hz variable-frequency drive. Wind velocity was measured with an S-type Pitot-tube anemometer, having a range of 0–80 m/s and a resolution of 0.01 m/s. The wind tunnel produced unidirectional, steady airflow (direction indicated in **Figure 1**, from left to right). Calibration under no-load conditions showed a velocity deviation of 7.7% and a turbulence intensity of 2.7%, meeting the experimental requirements.

### 2.3 Experimental treatments

Twelve treatment groups were established, with the control (CK) representing no wind. The six wind alone treatments were graded as follows: a, 3 m/s; b, 5 m/s; c, 7 m/s; d, 9 m/s; e, 11 m/s; f, 13 m/s. The five sand-laden treatments were: B, 5 m/s; C, 7 m/s; D, 9 m/s; E, 11 m/s; F, 13 m/s (The 3 m/s condition was excluded from the sand-laden treatments as it did not reach the saltation threshold required to initiate sand movement). Given the short, pulsed characteristics of the wind, treatments were limited to 10 minutes for each group.

The wind tunnel parameters were adjusted, and wind speeds were monitored in real time. Once the target wind speed was reached and stabilized, the seedlings were positioned in a straight line perpendicular to the airflow at the outlet and secured in place, after which the timer was started. At the end of the predetermined exposure period, the wind tunnel was powered off. To simulate sand-laden wind, a bed of sandy soil, 1.3 m long and 10 cm deep, was placed at the front end of the test section.

The response of plants to stress often lags. Therefore, following treatment completion, direct sunlight was avoided, and adherent sand particles were removed from the leaf surface, and the plants were allowed to recover for one hour before sampling. Due to the varying sensitivities of different plant organs to wind, with leaves being the most susceptible, only leaves were harvested. A completely randomized design was employed: all mature leaves from each treatment group were collected and pooled within the group. Each parameter was measured using at least three replicate samples, each weighing 0.2 g. Fresh leaves were used to assess electrical conductivity and malondialdehyde (MDA) content, while the remaining leaves were flash-frozen in liquid nitrogen and stored at −80 °C for subsequent analysis of physiological indicators.

### 2.4 Measurement indicators and methods

#### 2.4.1 Measurement of physiological parameters

Photosynthetic parameters were measured on a sunny morning using a Li-6800 (Li-Cor, USA) portable photosynthesis system. After treatment completion, the third mature leaf from the top was selected, avoiding the veins, and the net photosynthetic rate (Pn), transpiration rate (Tr), stomatal conductance (Gs), and intercellular CO_2_ concentration (Ci) of the seedlings were measured in a stable environment.

Membrane permeability was assessed using the electrical conductivity method with a portable conductivity meter (Model DDSJ-308A, INESA Scientific Instrument Co., Ltd., Shanghai, China), while MDA levels were determined via the thiobarbituric acid (TBA) assay. The activities of superoxide dismutase (SOD), peroxidase (POD), and catalase (CAT) were measured using nitroblue tetrazolium (NBT) staining, the guaiacol colorimetric method, and ultraviolet (UV) absorption, respectively, while proline contents were quantified using the acidic ninhydrin method, and soluble protein contents were determined using Coomassie Brilliant Blue (CBB), and soluble sugar content was performed using the anthrone colorimetric method (ACM) (see Roger, 2001 for details).

#### 2.4.2 Morphological assessments

Based on the results of preliminary experiments, the mechanical damage inflicted by sand-laden wind stress on the seedlings was further investigated. Three different treatments were applied: no wind (CK), wind alone at 11 m/s (e), and sand-laden wind at 11 m/s (E). Following treatment and a 1-hour recovery period, visible damage was assessed and photographed. Leaf samples were randomly collected from visibly damaged areas, with comparable positions selected across all seedlings. The samples were rinsed in phosphate−buffered saline (PBS) (pH 6.8), fixed with an electron microscopy−grade fixative (2.5% glutaraldehyde) for 4 hours, and then observed and imaged under a scanning electron microscope (SU8100-Japan). Following damage assessment, the seedlings were maintained for another three days to assess their ability to resume growth.

Four indicators were selected for statistical assessment: (1) Rate of leaf damage, defined as the proportion of damaged leaves relative to the total number of functional leaves, with leaves that remained abnormal after the recovery period classified as damaged; (2) Rate of trichome loss, calculated immediately after treatment as the number of trichomes per unit stem length in treated seedlings relative to that in the wind-free control; (3) Proportion of abnormal stomata, defined as the ratio of obstructed or deformed stomata to the total number of stomata within randomly selected high-magnification fields of view; counts were performed directly due to the low stomatal density; (4) Rate of wax layer loss, measured as the area of wax−layer exfoliation relative to the total area within a randomly selected high−magnification field of view.

#### 2.4.3 Measurement of plant hormones

The levels of IAA, GA, Zeatin, ABA, JA, and SA were determined using an Agilent 1290 HPLC system coupled to a QTRAP 6,500 mass spectrometer (SCIEX). LC-MS/MS analysis was performed by Nanjing Webiolotech Testing Technology Co., Ltd. (Nanjing, China). To correct for matrix effects and losses during sample preparation, isotope-labeled internal standards—including deuterated IAA (D-IAA), ABA (D-ABA), JA (D-JA), SA (D-SA), Zeatin (D-TZeatin), and GA₄ (D-GA₄)—were added to each sample prior to extraction (all sourced from Shanghai Zhenzhun Biotechnology Co., Ltd., China). Detailed mass spectrometry parameters (e.g., multiple reaction monitoring transitions) are provided in **Supplementary Table 1**. Hormone content (ng/g) was calculated as the detected concentration (ng/mL) multiplied by the final volume (mL) and divided by the sample mass (g).

### 2.5 Statistical analysis

Data were analyzed using Microsoft Excel and SPSS Statistics 27.0.1 (IBM Corp., Armonk, NY, USA) and are presented as mean ± standard error (SE) from at least three biological replicates (n ≥ 3). Statistical differences among treatments were analyzed using one-way ANOVA followed by LSD *post-hoc* test (p < 0.05). Figures were generated using Origin 2024 (OriginLab Corporation, Northampton, MA, USA) and R software (R Foundation for Statistical Computing, Vienna, Austria). Correlation and principal component analyses were performed via Origin 2024. Multiple Factor Analysis (MFA) was conducted using the “FactoMineR” package in R-4.5.1.

## 3 Results

### 3.1 Effect of wind alone and sand-laden wind on physiological parameters

#### 3.1.1 Photosynthetic parameters

**Figure 2:**
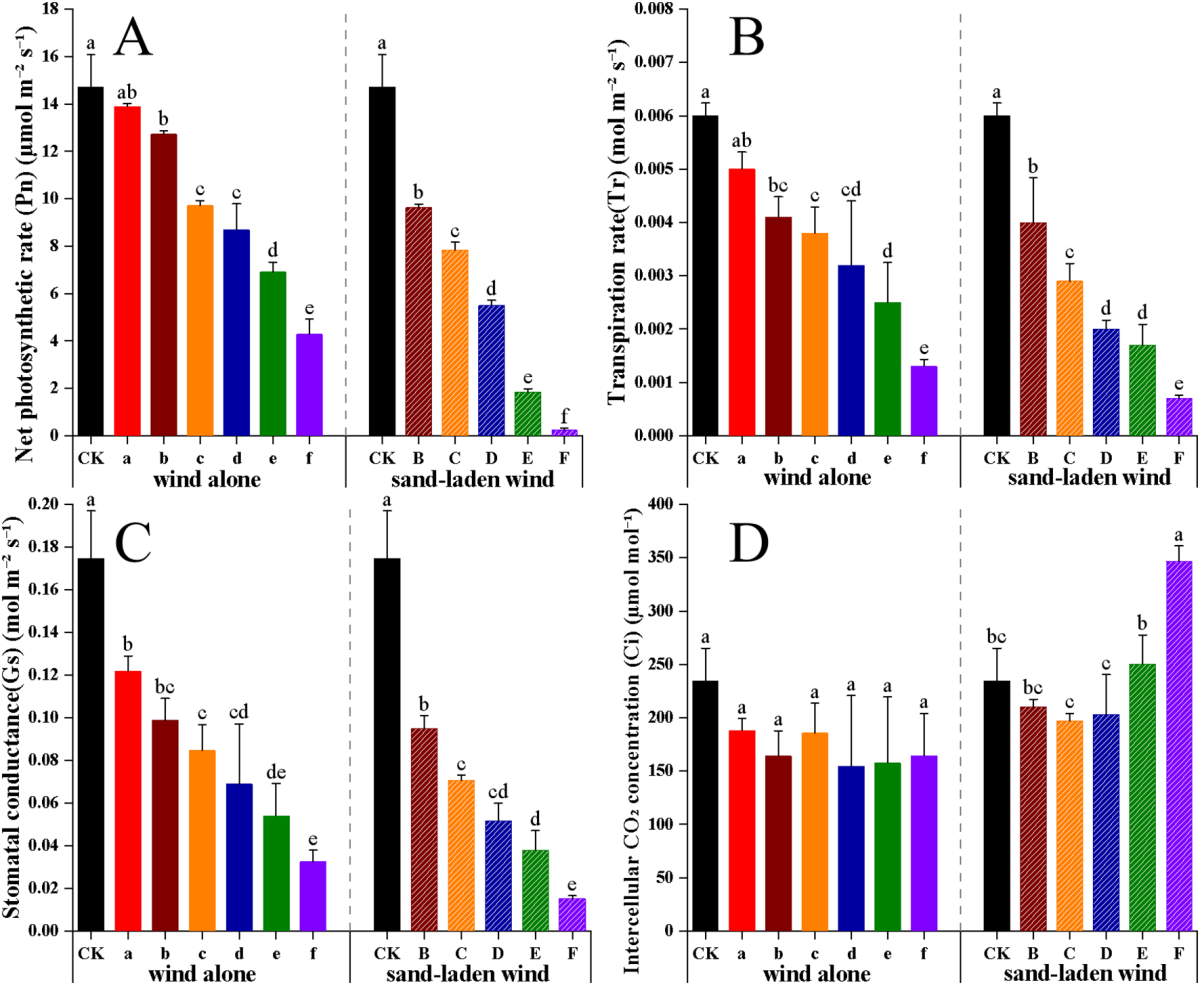
Effects of wind alone and sand-laden wind on photosynthetic parameters of tomato seedlings. **(A)** Net photosynthetic rate (Pn), **(B)** Transpiration rate (Tr), **(C)** Stomatal conductance (Gs), and **(D)** Intercellular CO_2_ concentration (Ci). Seedlings were exposed to control (CK, no wind), wind alone (3–13 m/s), or sand-laden wind (5–13 m/s) for 10 min. Data are presented as mean ± standard error (SE) from three biological replicates (n = 3). Statistical differences among all treatments were analyzed using one-way ANOVA followed by a least significant difference (LSD) *post-hoc* test. Different lowercase letters above the bars indicate statistically significant differences at p < 0.05.

The Pn, Tr, and Gs values of the tomato seedlings all declined significantly with the increase in intensity of the wind alone and sand-laden wind. At the same wind speed, the reductions were more pronounced under sand-laden wind treatment (**Figure 2**). Under sand-laden wind treatment F, net Pn, Gs, and Tr decreased by 98%, 91%, and 88%, respectively, compared with the CK, resulting in nearly complete inhibition of photosynthesis. Under wind alone treatment at the same speed (treatment f), the corresponding reductions were 71%, 81%, and 78%, respectively. The intercellular CO_2_ concentration (C_i_) did not decrease significantly under wind alone treatment (*p* = 0.3); the lowest recorded value was 154.5 μmol/mol following treatment d, which was 34% lower than the value observed for CK (234.4 μmol/mol). Under sand-laden wind conditions, intercellular Ci initially decreased but increased significantly (*p <* 0.05) at wind speeds exceeding 7 m/s (treatment C), reaching a maximum in treatment F that was 48% higher than CK. Both wind alone and sand-laden wind treatments significantly suppressed photosynthetic parameters, with the exception of Ci. Sand-laden wind exerted a stronger inhibitory effect on photosynthesis than wind alone, and the magnitude of inhibition increased with wind speed.

#### 3.1.2 Electrical conductivity and MDA levels

**Figure 3:**
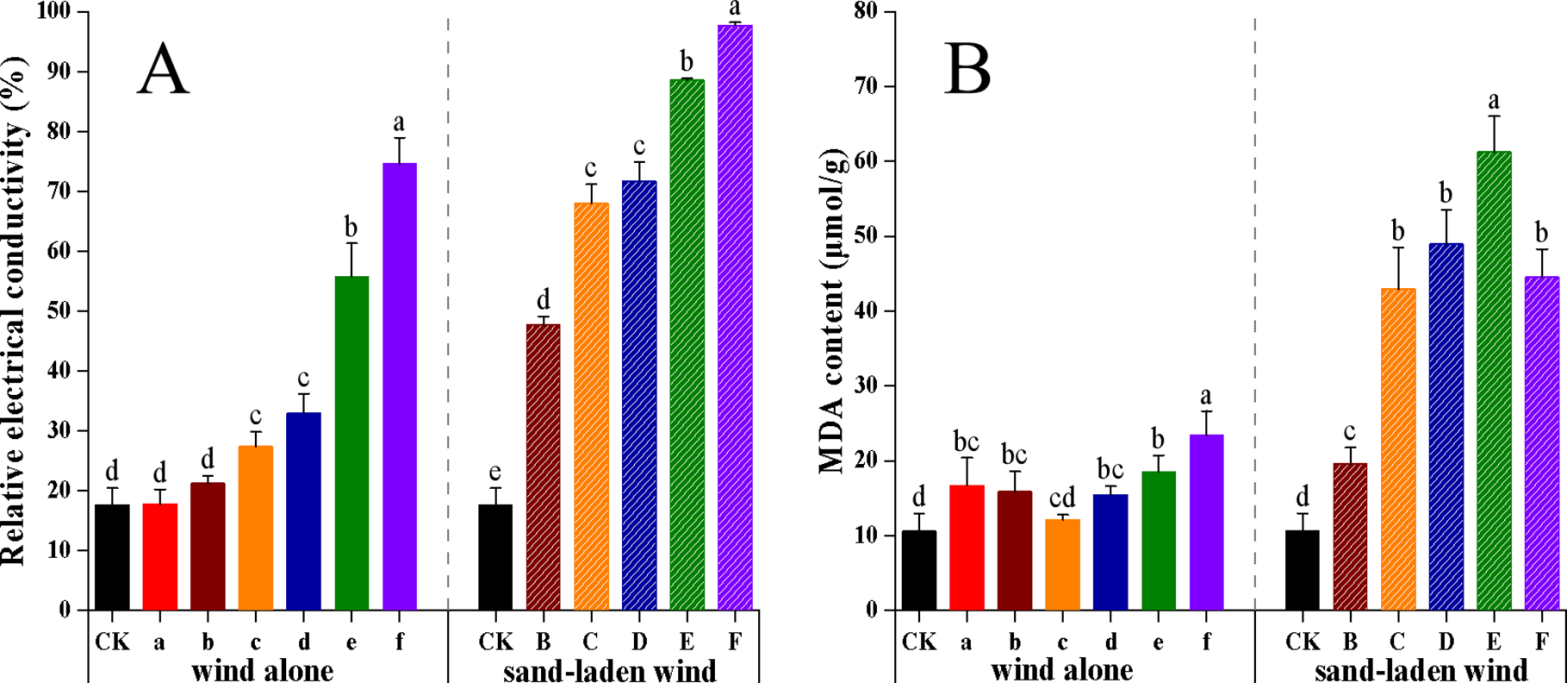
Effect of wind alone and sand-laden wind on the membrane system of tomato seedlings. **(A)** Relative electrical conductivity; **(B)** MDA content. Seedlings were exposed to control (CK, no wind), wind alone (3–13 m/s), or sand-laden wind (5–13 m/s) for 10 min. Data are presented as mean ± standard error (SE) from three biological replicates (n = 3). Statistical differences among all treatments were analyzed using one-way ANOVA followed by a least significant difference (LSD) *post-hoc* test. Different lowercase letters above the bars indicate statistically significant differences at p < 0.05.

Both wind alone and sand-laden wind treatments led to increased relative electrical conductivity and MDA levels in seedlings, exhibiting a stronger upward response under sand-laden wind (**Figure 3**). Under wind alone treatment, the relative electrical conductivity initially increased slowly, then rose rapidly as the wind speed increased, ultimately reaching a maximum of 75%. Under sand-laden wind treatment, relative electrical conductivity increased significantly throughout (*p* < 0.001), finally peaking at 97%. At each equivalent wind speed, conductivity values were substantially higher following sand-laden wind treatment than after wind alone treatment. Regarding MDA contents, wind alone treatment caused an initial increase, followed by a decline and a subsequent rise, with all values remaining above CK levels. The highest MDA content under wind alone conditions was 23.4 μmol/g, representing a 121% increase over CK (10.6 μmol/g). Under sand-laden wind treatment, MDA content increased significantly with treatment intensity (*p <* 0.001), peaking at 61.2 μmol/g at 11 m/s (Treatment E) before declining, corresponding to a 478% increase relative to CK. These results indicate that both wind alone and sand-laden wind disrupted the membranes of tomato seedlings, with membrane damage and associated oxidative stress being markedly more severe under sand-laden wind than wind alone.

#### 3.1.3 Activities of antioxidant enzymes

**Figure 4:**
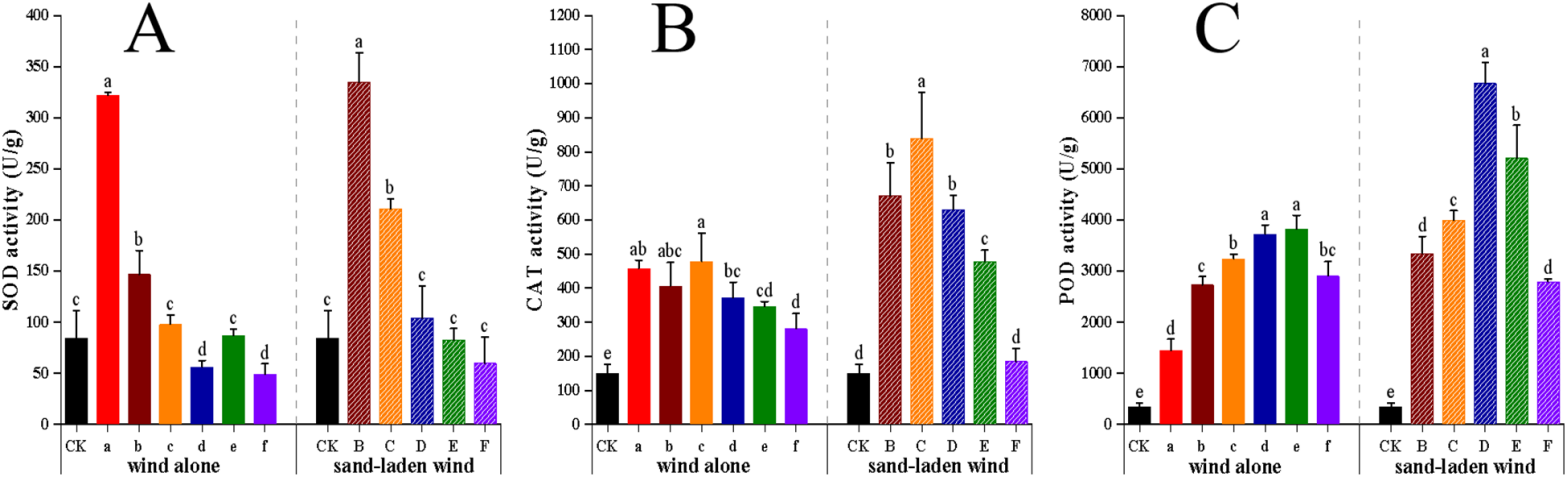
Effect of wind alone and sand-laden wind on antioxidant enzyme activities of tomato seedlings. **(A)** SOD activity; **(B)** CAT activity; **(C)** POD activity. Seedlings were exposed to control (CK, no wind), wind alone (3–13 m/s), or sand-laden wind (5–13 m/s) for 10 min. Data are presented as mean ± standard error (SE) from three biological replicates (n = 3). Statistical differences among all treatments were analyzed using one-way ANOVA followed by a least significant difference (LSD) *post-hoc* test. Different lowercase letters above the bars indicate statistically significant differences at p < 0.05.

Both wind alone and sand-laden wind significantly increased the activities of the antioxidant enzymes, SOD, CAT, and POD. Overall, the activities of all three enzymes increased initially before decreasing as the wind speed intensified, with the response following sand-laden wind treatment being stronger than that after wind alone treatment at the same wind speed (**Figure 4**). SOD activity was observed to peak after treatment with the lowest intensities of both wind alone and sand-laden winds, with peak values of 321 U/g and 334.7 U/g, respectively, reflecting the sensitivity of this enzyme to oxidative stress. CAT activity showed higher peak values: 478 U/g after exposure to 7 m/s wind alone and 839.2 U/g to 7 m/s of sand-laden wind. Apart from that observed at the 13 m/s wind speed, CAT activity was consistently higher following sand-laden wind treatment than wind alone treatment, with significantly more pronounced between−group differences under sand-laden wind (*p* < 0.01), indicating that CAT is more sensitive to sand-laden wind. The highest activity among the three enzymes was seen for POD, which peaked at 3816 U/g under 11 m/s wind alone and at 6670 U/g under 9 m/s sand-laden wind, representing increases of 1,009% and 1,839%, respectively, compared to CK. The activities of the three enzymes peaked in succession as the intensity of the wind stress increased, with the change amplitude of POD activity far exceeding that of the other two enzymes, playing a dominant role in the response process.

#### 3.1.4 Proline, soluble sugar, and soluble protein levels

**Figure 5:**
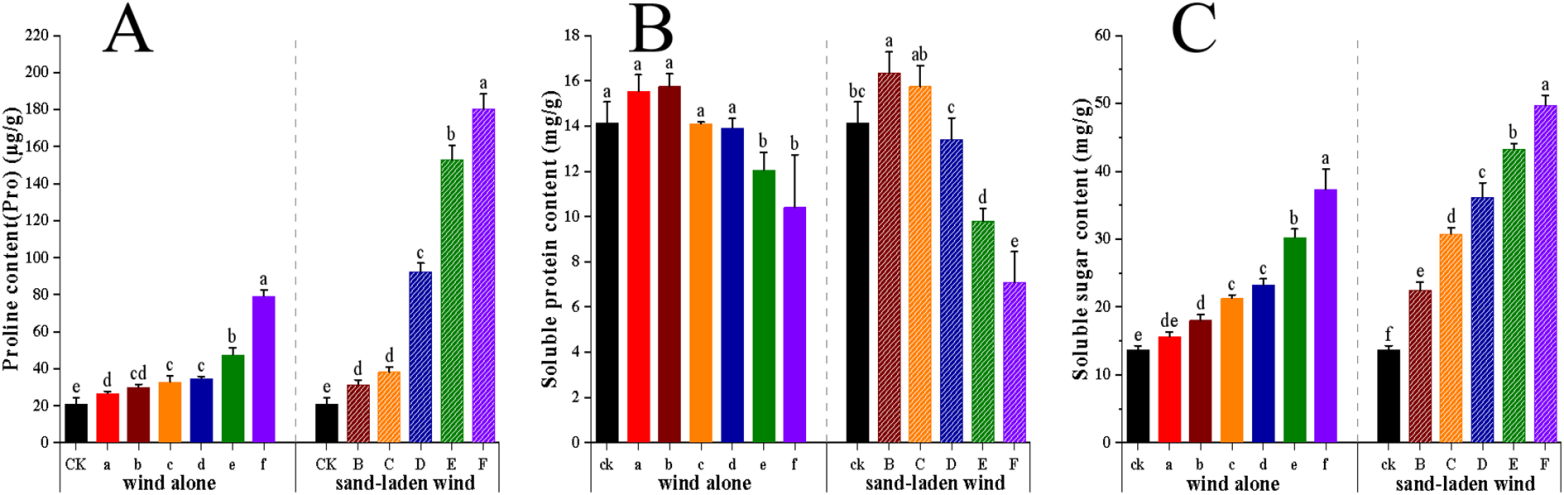
Effect of wind alone and sand-laden wind on osmotic adjustment capacity of tomato seedlings. **(A)** Proline content; **(B)** Soluble protein content; **(C)** Soluble sugar content. Seedlings were exposed to control (CK, no wind), wind alone (3–13 m/s), or sand-laden wind (5–13 m/s) for 10 min. Data are presented as mean ± standard error (SE) from three biological replicates (n = 3). Statistical differences among all treatments were analyzed using one-way ANOVA followed by a least significant difference (LSD) *post-hoc* test. Different lowercase letters above the bars indicate statistically significant differences at p < 0.05.

Under both wind alone and sand-laden wind treatments, proline (Pro) and soluble sugar contents increased continuously with the intensity of the wind speed (*p* < 0.01), while soluble protein levels first rose and then declined. The amplitude of change after sand-laden wind treatment was greater than that following wind alone treatment at the same wind speed (**Figure 5**). Specifically, the Pro content increased slowly under wind alone, with a maximum rise of 279% relative to CK. Under sand-laden wind, Pro levels increased significantly (*p* < 0.01) when the wind intensity exceeded 7 m/s (treatment C), reaching a maximum increase of 765%. The soluble sugar content showed a similar trend to that of Pro, increasing significantly throughout (*p* < 0.01), with peak accumulation under the two treatments rising by 172% and 263%, respectively, compared to CK. Soluble protein levels did not differ significantly (*p* > 0.05) between 3 and 9 m/s wind alone (treatments a–d), but decreased markedly beyond 11 m/s of sand-laden wind (treatment E), with the lowest value being 26% below that of CK. Under sand-laden wind, soluble protein levels declined linearly from 7 m/s (treatment C) onward, with the lowest value being 50% below that of CK. These results indicate that sand-laden wind affects the plant’s osmotic balance to a greater extent than wind alone. Both Pro and soluble sugars play a dominant role in this process. Under high-intensity wind-sand stress, both show substantial accumulation, accompanied by the degradation of soluble protein.

#### 3.1.5 Comprehensive assessment of the physiological impact of wind alone and sand-laden wind treatments on seedlings

The effects of different treatment intensities encompass multidimensional data that cannot be accurately represented by any single indicator. Therefore, the different physiological data were standardized and then integrated using correlation analysis and principal component analysis (PCA). A composite score was calculated based on the scores and weightings of each treatment on the two principal components, to reflect the stability of the physiological system.

**Figure 6:**
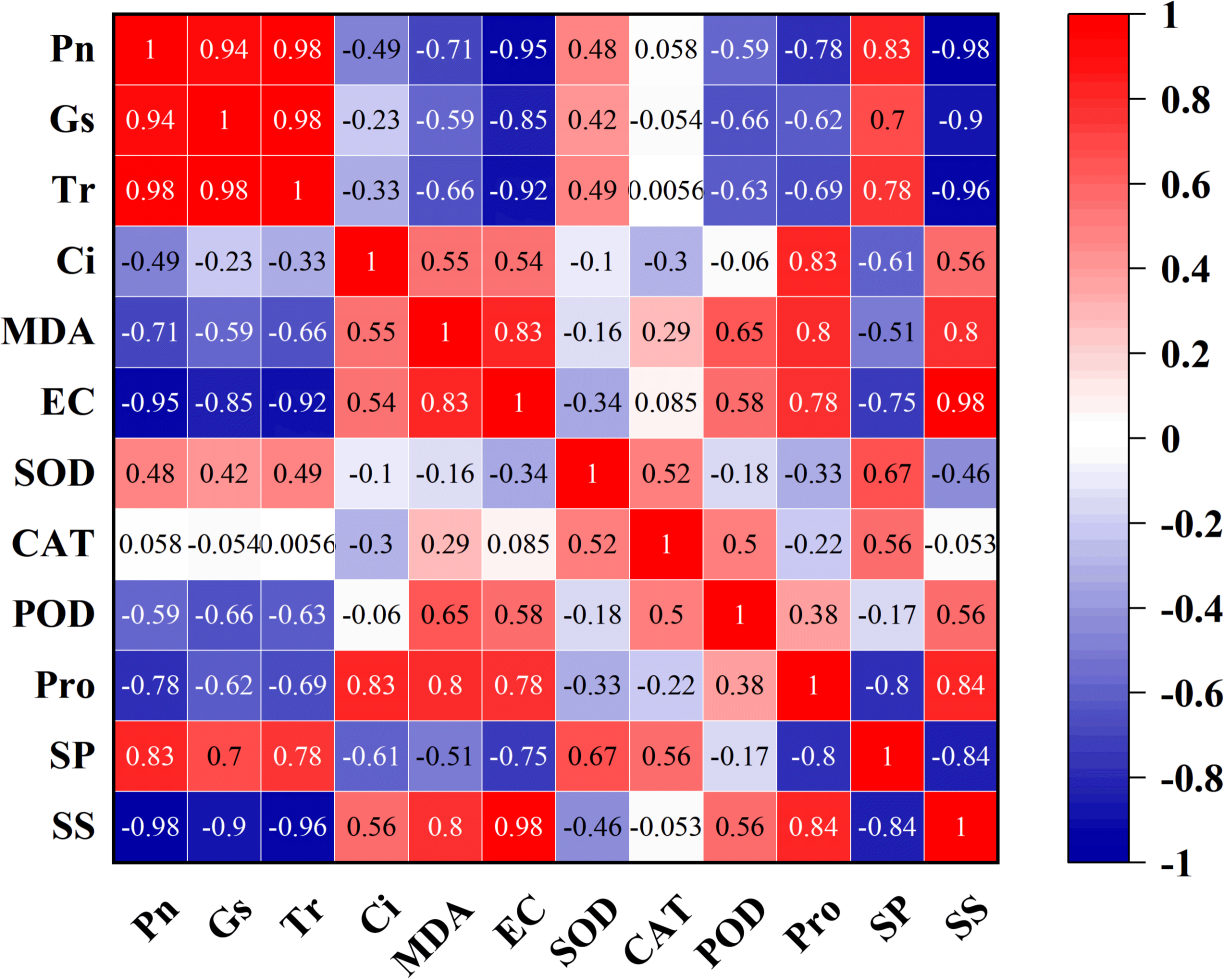
Heatmap of correlations among different physiological parameters of tomato seedlings under wind alone and sand-laden wind stress. The color scale represents Pearson correlation coefficients, ranging from -1 (strong negative correlation, blue) to +1 (strong positive correlation, red).

As shown in **Figure 6**: Pn, Gs, and Tr were strongly positively correlated (correlation coefficients ≈ 1), indicating that they varied in close synchrony. This synchronization reflects the central regulatory role of stomatal behavior in the photosynthetic process. In addition, stress-related indices, such as MDA, relative electrical conductivity, Pro, and soluble sugar, were also strongly and positively associated, illustrating the coordinated response of the membrane and osmotic adjustment systems. The photosynthetic indices showed strong negative correlations with all other stress indicators (correlation coefficients ≈ -1), demonstrating the close association between reduced photosynthetic capacity and increased stress. The results of the correlation analysis confirm that under both wind alone and sand-laden wind stress, the photosynthetic capacity of the seedlings decreases, accompanied by membrane damage, oxidative injury, and osmotic stress.

**Figure 7:**
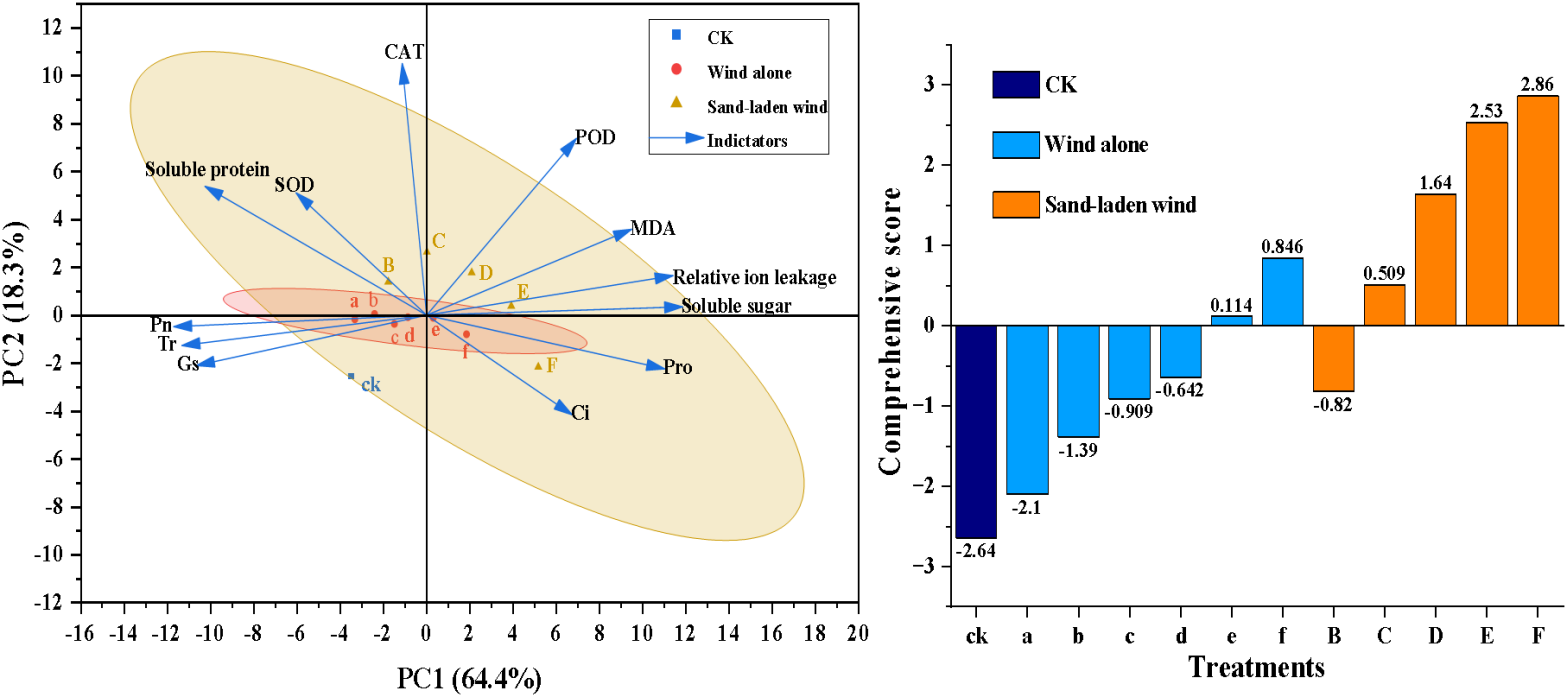
Principal component analysis (PCA) of all physiological indicators under different treatments. (Left) Score plot of treatments on the first two principal components (PC1 and PC2). The percentage of variance explained by each component is shown in parentheses. (Right) Comprehensive PCA scores of treatments, calculated based on the weighted scores of PC1 and PC2 according to their contribution to total variance. A higher composite score indicates greater deviation from the control (CK) and a more severe stress response.

The PCA results showed that principal components PC1 and PC2 explained 64.4% and 18.3% of the variance, respectively, with a cumulative explanation rate of 82.7%, indicating that the first two principal components provided an effective reflection of the differences in physiological indicators among the treatments. The positive direction of PC1 was associated with MDA, relative electrical conductivity, soluble sugars, and Pro, all of which are associated with membranes and osmotic regulation, while the negative direction included Pn, Gs, and Tr, related to photosynthesis. The positive direction of PC2 was primarily related to the antioxidant enzymes (**Figure 7**, left). The distribution of treatments reflects their correlation with each principal component. The wind alone treatments clustered mainly along the negative direction of PC1, indicating that wind alone primarily led to reduced photosynthesis and osmotic stress, with evidence of membrane damage apparent at high wind speeds. In contrast, sand-laden wind treatments showed pronounced spatial separation along both principal components, suggesting the plant’s response to these conditions involved all physiological systems and was more intense and complex than that to wind alone.

Using CK as the baseline, a lower composite PCA score indicates a more stable physiological state. Within each series of treatments, the composite score increased consistently as the level of stress rose (**Figure 7**, right), demonstrating positive correlations between the magnitude of the physiological response and the intensity of the stress. Under wind alone treatment conditions, the composite score for treatment e shifted from negative to positive, indicating that wind alone at speeds of 11 m/s or higher may exceed the self−regulatory capacity of the plant, resulting in clear damage to the seedlings. Similarly, under sand-laden wind treatment, treatment C was found to be the turning point, suggesting that sand-laden wind at 7 m/s may already lead to marked seedling damage. This confirms that sand-laden wind induces higher levels of stress than wind alone.

### 3.2 Effect of wind alone and sand-laden wind stress on morphological characteristics of tomato seedlings

After exposure to severe stress, various physiological indicators exhibited abnormal responses that deviated from the overall trend, such as increased intercellular CO_2_ concentration, decreased enzyme activity, and reduced soluble protein content under the 13 m/s sand-laden wind treatment. These anomalies were attributed to the intensity of the stress exceeding the self−regulatory capacity of the seedlings, with severe mechanical damage leading to the dysfunction of physiological homeostasis. To verify whether these abnormal physiological changes were associated with mechanical injury, two markedly contrasting treatment groups, e and E, were selected for further investigation, beginning with an assessment of morphological damage.

**Figure 8:**
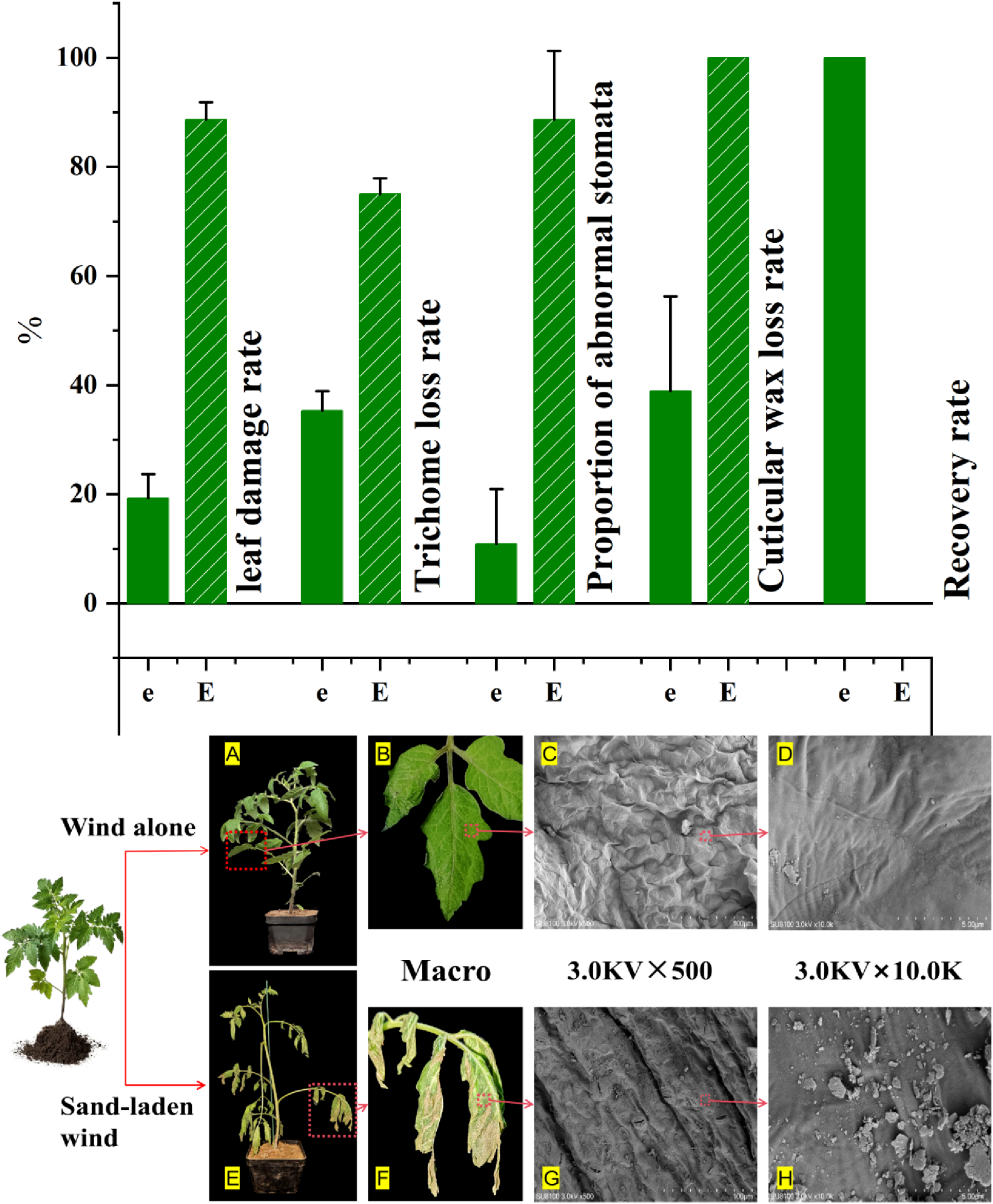
Morphological changes in tomato seedlings from the whole-plant to micro-structural level following 10 min high-intensity wind alone (11 m/s) and sand-laden wind (11 m/s) treatments. **(A, E)** Whole-plant morphology at one hour after treatment. **(B, F)** Representative images of leaf damage. **(C, D, G, H)** Scanning electron microscope (SEM) images of leaf surfaces. Quantitative assessments are presented as percentages for the following parameters: (I) leaf damage rate, (II) trichome loss rate, (III) proportion of abnormal stomata, (IV) cuticular wax loss rate, and (V) recovery rate of seedlings after a three-day recovery period. For quantitative data (I-V), values represent means ± SE (n = 3 biological replicates).

Wind alone treatment caused relatively minor damage to the tomato seedlings, primarily manifesting as water stress, with significant damage occurring only at higher wind speeds. After a three-day recovery period, growth largely resumed, the recovery rate is 100%. Following exposure to wind alone at 11 m/s, the seedlings appeared healthy, with mostly intact leaves showing no evidence of dust adhesion (**Figure 8A**). The leaf damage rate was approximately 19%, while trichome loss reached 35% (**Figure 8B**). Epidermal cells were uniformly dense with smooth surfaces, although signs of dehydration-induced shrinkage were observed (**Figure 8C**). Stomata were fully closed, exhibiting clear and regular apertures with no abnormal attachments; the proportion of abnormal stomata was approximately 11%, with most retaining normal function. The wax layer exhibited slight peeling in some areas, with a loss rate of about 39% (**Figure 8D**). Damage to the wax layer can increase water loss, thus exacerbating water stress.

The sand-laden wind treatment resulted in more pronounced effects on seedling morphology compared to the wind alone treatment. After sand-laden wind treatment at 11 m/s, the tomato seedlings exhibited pronounced mechanical damage, primarily in the form of leaf tearing and petiole breakage. Following the recovery period, all affected functional leaves withered and failed to resume growth, the recovery rate is 0%. The overall condition of the seedlings was poor, with evident lodging (**Figure 8E**). The leaves were torn and damaged, with dried and wilted edges, while large brown patches were apparent on the surface, with fine sand particles adhering to the surface (**Figure 8F**). The rate of leaf damage was 89%, while the rate of trichome loss was 75%. The cells appeared shrunken and deformed, with fractured margins. Most stomata were blocked and covered by sand particles, exhibiting distorted apertures and were unable to close. The proportion of abnormal stomata was 89% (**Figure 8G**), suggesting severely impaired gas exchange and water regulation. The leaf epidermis was rough, with complete (100%) loss of the wax layer. Numerous particles (1–10 μm in diameter) adhered to or became embedded in the epidermis (**Figure 8H**), increasing the risk of water loss and susceptibility to pathogen invasion.

### 3.3 Effect of mechanical damage on endogenous hormones in tomato seedlings

Following the assessment of morphological damage, the tomato seedlings were further sampled and analyzed. Changes in levels of common endogenous hormones (IAA, GA, zeatin, ABA, JA, SA) were determined one hour after treatment to evaluate hormonal regulation in response to mechanical damage.

**Figure 9:**
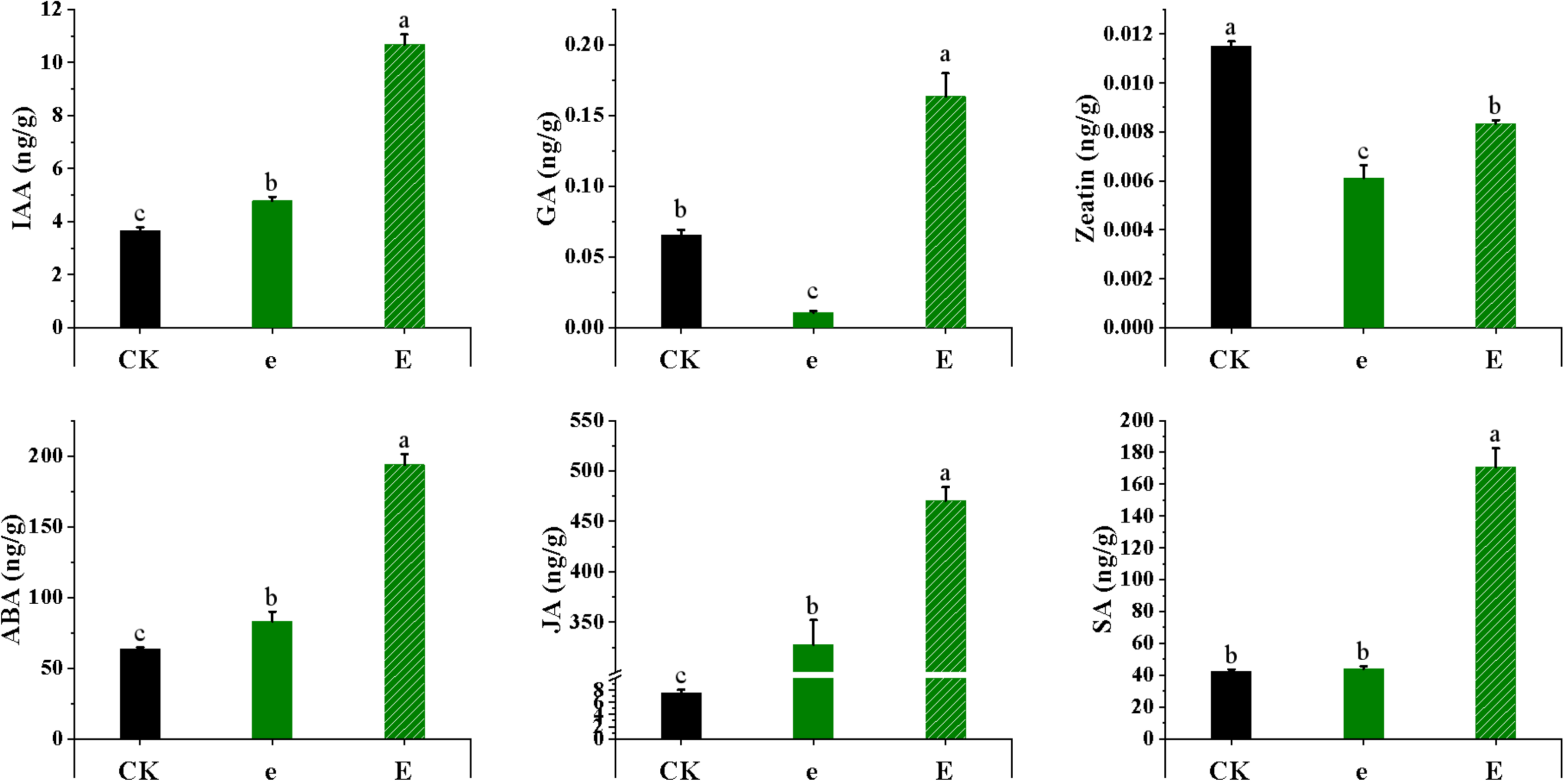
Effect of high-intensity wind alone (11 m/s) and sand-laden wind (11 m/s) on key phytohormones in tomato leaves. Concentrations of IAA, GA, Zeatin, ABA, JA, and SA were measured one hour after treatment using LC-MS/MS. Values are means ± SE (n = 3 biological replicates). Statistical differences among treatments for each hormone were analyzed using one-way ANOVA followed by LSD *post-hoc* test. Different letters above the bars indicate significant differences at p < 0.05.

As shown in **Figure 9**: Overall, hormone levels following the sand-laden wind treatment were higher than those after the wind alone treatment, with the levels of defense-related hormones (ABA, JA, SA) substantially exceeding those of growth-related hormones (IAA, GA, Zeatin). Compared with CK, the wind alone treatment increased the contents of IAA, ABA, JA, and SA by 30.9%, 30.5%, 4,231.8%, and 5%, respectively. Under sand-laden wind treatment, the corresponding increases were 191.5%, 205%, 6,126.3%, and 302.6%. GA levels decreased by 83.6% following wind alone treatment but increased by 150% under sand-laden wind. Zeatin content declined by 49.1% and 30.8% under wind alone and sand-laden wind conditions, respectively, indicating that although zeatin decreased in both treatments, it remained relatively higher under sand-laden wind than under wind alone. These hormonal changes indicate that sand-laden wind induces more severe stress than wind alone, resulting in greater accumulation of defense-related hormones and suppressed production of growth-related hormones. Mechanical injury specifically triggers a pronounced activation of the systemic defense signaling network in plants, particularly affecting the synthesis and signaling of key hormones such as ABA, JA, and SA, suggesting that plants mobilize stronger defense mechanisms in response to mechanical damage.

### 3.4 Comprehensive evaluation of the overall impact of wind alone and sand-laden wind treatments on seedlings

Evaluate the comprehensive impact of physiological indicators, morphological indicators, and hormone content on tomato seedlings using multidimensional factor analysis (MFA) (Visbal-Cadavid et al., 2020).

**Figure 10:**
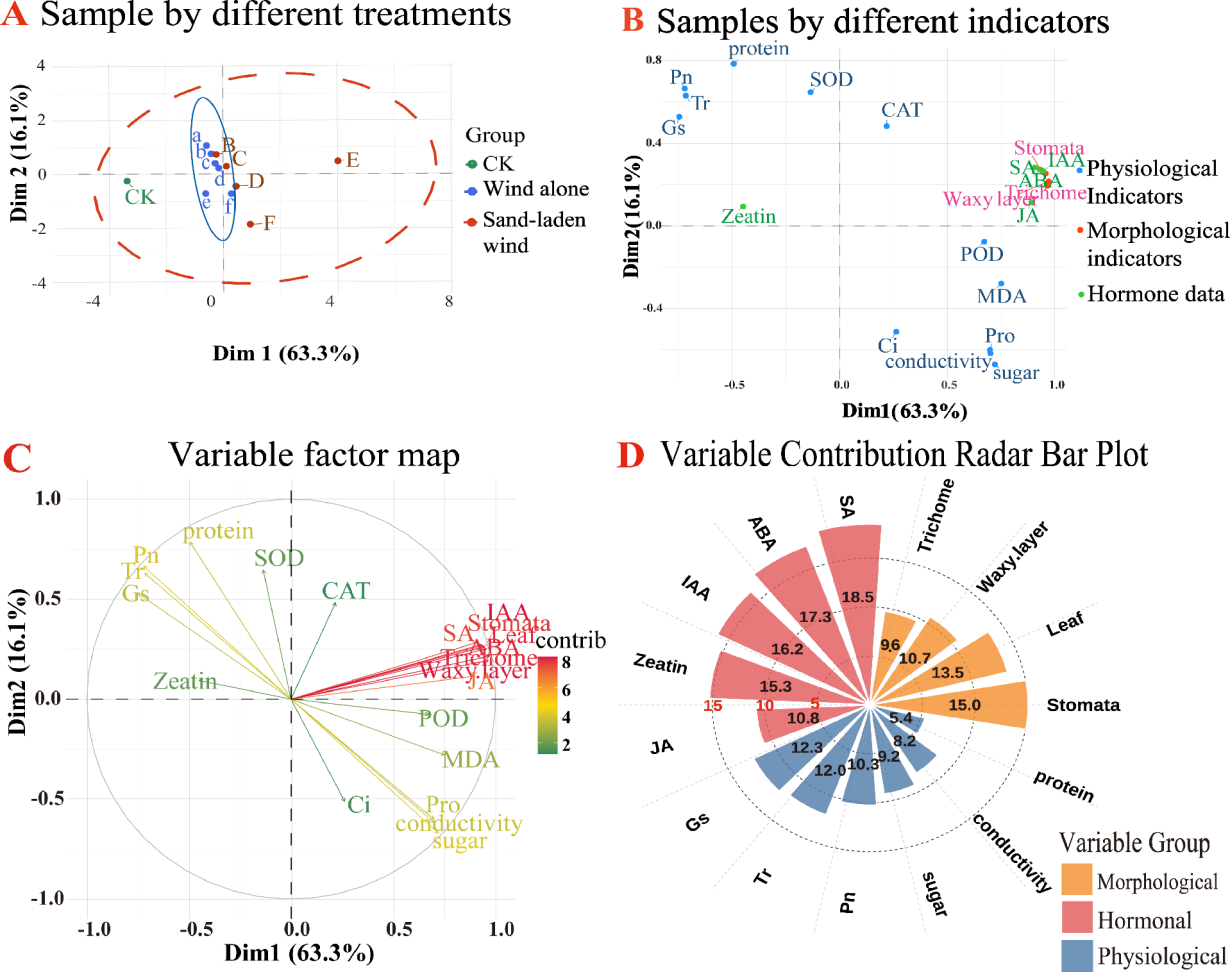
Multiple factor analysis (MFA) integrating physiological, morphological, and hormonal indicators to evaluate the overall impact of wind alone and sand-laden wind on tomato seedlings. **(A)** Scatter plot of MFA samples under different treatments; **(B)** Scatter plot of MFA samples by different indicators; **(C)** MFA variable factor map by different indicators; **(D)** MFA contribution distribution map by different indicators.

The first two dimensions explained 63.3% and 16.1% of the total variance, respectively, cumulatively accounting for 79.4%. Samples from the wind alone treatments were observed to cluster mainly near Dim2, whereas those from the sand-laden wind treatments were more dispersed. Morphological indicators showed the most concentrated distributions, clustering around Dim1 together with the hormone data, while the physiological indicators were more scattered and were more strongly associated with Dim2 (**Figures 10A, B**). These findings indicate that wind alone treatment was associated most strongly with physiological indicators, while sand-laden wind treatment induced substantial changes in physiological indicators, morphological traits, and hormone contents. **Figures 10C, D** further show that the measurements of hormones and morphological indicators contributed most significantly to the comprehensive evaluation, constituting core variables for distinguishing damage caused by wind alone and sand-laden wind. The MFA results demonstrate that wind alone stress tends to affect the physiological systems of the seedlings, whereas sand-laden wind induces multiple interrelated changes among physiological indicators, morphological traits, and hormone levels. Among these, the hormone levels provided the best reflection of the impact of sand-laden wind on tomato seedlings, followed by morphological indicators, and lastly, physiological indicators. These results suggest that mechanical damage is a primary cause of the effects exerted by sand-laden wind on tomato seedling growth.

## 4 Discussion

Under conditions of mild stress, both wind alone and sand-laden wind treatments accelerated water loss by disruption of the leaf microenvironment. The observed decline in Gs suggests ABA-mediated stomatal closure, a common response under water stress induced by wind (Lee and Luan, 2012). This stomatal closure subsequently reduces Tr and restricts CO_2_ diffusion, resulting in decreased Ci and Pn. This stage was thus primarily characterized by stomatal limitations (Zeng et al., 2024). Under conditions of sand-laden wind stress, the decrease in Pn, Gs, and Tr was observed to be significantly greater than that under wind alone treatment, indicating that the presence of sand particles not only exacerbated water stress but also likely interfered directly with the photosynthetic system (for example, blocking stomata). This effect was immediately reflected in the membrane system, manifested as increases in the relative electrical conductivity and MDA content, with the magnitude of change under sand-laden wind treatment being significantly larger than that under wind alone treatment at the same wind speed. These findings demonstrate that the presence of sand particles intensifies stress, resulting in membrane damage and lipid peroxidation (Lv et al., 2024). The morphological indicators, such as dehydration shrinkage and leaf damage, also partially corroborate this conclusion. In response to reactive oxygen species (ROS) generated due to membrane system damage, the antioxidant enzyme system was rapidly activated. SOD activity was found to peak first, reflecting the sensitivity of the enzyme to oxidative stress (Mittler, 2002), followed by successive increases in POD and CAT activities, with POD consistently playing the dominant role. The osmotic adjustment system was also activated to maintain cell turgor and water balance, leading to the continuous accumulation of Pro and soluble sugars, which was observed to be more pronounced following sand-laden wind treatment. These changes align with patterns reported under various other plant stress conditions. It is therefore hypothesized that during the initial or mild stress phase, plants activate a conserved stress−response program, and sand-laden wind amplifies every phase of this program through the combined effects of water stress and sand-particle impact.

When stress exceeds a critical threshold, mechanical damage ensues. If the damage is severe enough, the physiological system shifts from adaptive responses to dysfunctional states, a shift that was particularly pronounced following sand-laden wind treatment. In other words, the threshold for seedling damage under sand-laden wind is substantially lower than that observed under wind alone treatment. Following severe sand-laden wind stress, the values of Pn, Gs, and Tr exhibited a marked decline, in contrast to Ci, which showed abnormal increases, reflecting a transition in the primary limiting factor from stomatal to non-stomatal constraints (Farquhar and Sharkey, 1982). Taken together with the observed leaf injury, stomatal deformation, and cellular damage, this indicates that the combination of strong wind and sand particles caused direct damage to the structure of mesophyll cells and the photosynthetic apparatus, resulting in a loss of carbon assimilation capacity. Mesophyll conductance has been reported to decline significantly upon damage to the cellular structure, blocking the diffusion of CO_2_ to carboxylation sites and causing a rapid drop in CO_2_ utilization efficiency (Lin et al., 2022). Respiratory activity has been reported to increase in damaged tissues to allow tissue repair and recovery (Sharkey, 2005). These processes lead to an accumulation of CO_2_ in intercellular spaces. At 13 m/s sand-laden wind stress, relative electrical conductivity neared 100%, signifying extensive membrane disruption. The observed reduction in MDA content presumably resulted from the arrest of lipid peroxidation after rapid cell death or from methodological interference caused by the efflux of cellular constituents (Halliwell and Gutteridge, 1986). Both phenomena are indicative of a severe disruption of physiological homeostasis.

Under both wind alone and sand-laden wind conditions, antioxidant enzyme activity rose to a maximum before declining significantly as stress intensity further increased. The decline does not reflect reduced demand for defense but is instead another manifestation of systemic dysfunction. Mechanical damage led to a rate of ROS generation that exceeded the plant’s scavenging capacity, leading to the degradation or impaired synthesis of the enzymes and, ultimately, to the failure of the antioxidant defense system (Gill and Tuteja, 2010). This resulted in damage that, based on the absence of visible recovery after three days, appeared to be irreversible at the whole-plant level. While osmoregulatory substances, such as proline, can also scavenge ROS and can provide a certain degree of protection after the collapse of the antioxidant enzyme system (Yu et al., 2017), their continuous accumulation under amplified stress factors occurred alongside significant degradation of soluble proteins. This may represent an early signal of carbon–nitrogen metabolic reprogramming. Under imbalances in carbon and nitrogen metabolism, the plant may reallocate resources by degrading functional proteins (e.g., Rubisco) to sustain the supply of precursors required for osmolyte synthesis (Nunes-Nesi et al., 2010; Araújo et al., 2011). This process is not a normal osmoregulatory response but an emergency reaction induced at the expense of fundamental metabolic functions. Moreover, proline synthesis consumes substantial amounts of NADPH, which can accelerate the collapse of the physiological system (Liu et al., 2008). It should be noted that the inference of “irreversible damage” and “system collapse” was based on the complete loss of membrane integrity at one hour after stress exposure and observations three days post-treatment. All sand-laden wind-treated seedlings exhibited complete leaf desiccation with no visible regrowth; however, stem turgor was maintained, preventing definitive assessment of whole-plant survival based on visual inspection alone. Although the lack of leaf recovery strongly suggests severe damage to photosynthetic tissues—damage that may lead to seedling death or loss of agronomic value—confirming the absolute irreversibility of this injury at the cellular or molecular level would require longer-term recovery studies. Such studies should include extended monitoring of biomass accumulation, new leaf emergence, and, if necessary, meristem viability staining to definitively assess whole-plant survival.

Plant hormones are essential signaling molecules that regulate plant development and responses to environmental stimuli (Zhu, 2016; Fàbregas and Fernie, 2021). In this study, the marked elevations in JA, ABA, and SA levels constitute the clearest evidence for elucidating the key regulators underlying sand-laden wind stress responses. JA, a key signaling molecule in the response to mechanical injury (Hu et al., 2013; Li et al., 2023), increased by 6,126.3% under 11 m/s sand-laden wind, offering the most direct molecular evidence that the presence of sand particles leads to severe mechanical damage. Increased levels were also observed following wind alone treatment, indicating that the treatment at 11 m/s already caused slight mechanical damage, consistent with the observations of minor leaf injuries and membrane damage. A 205% rise in ABA reflects the heightened water stress induced by sand-laden wind. ABA-mediated stomatal closure (Lee and Luan, 2012) led directly to the reductions in Gs, Tr, and Pn. Furthermore, synergy between ABA and JA amplified the regulation of downstream defense genes (Basu et al., 2025), highlighting the combined effect of water stress and mechanical damage under the influence of sand-laden wind. SA, which typically mediates defense responses against pathogens (Durrant and Dong, 2004), increased by 5% under wind alone treatment and by 302.6% under sand-laden wind. These elevations likely reflect inadvertent activation of the SA signaling pathway by damage-associated molecular patterns (DAMPs), such as cellular fragments released upon membrane rupture (Choi and Klessig, 2016). This unexpected activation may lead to crosstalk with the JA and ABA signaling pathways, marking an early shift in defense signaling from localized regulation toward a broader, systemic response. Although resource allocation was not directly quantified, this hormonal reprogramming is consistent with the initiation of a growth–defense trade-off, in which resources are likely redirected from growth toward defense and repair (de Vries et al., 2017).

Under stress, the growth–defense trade-off has been reported to downregulate growth-related hormone signaling (Huot et al., 2014), which is highly consistent with the observed decreases in IAA, GA, and Zeatin levels in the present study. IAA as a core hormone that controls plant organ formation and cell elongation, and also plays a leading role in the reconstruction of vascular tissues during wound repair (Matsuoka et al., 2016). Its greater increase following sand-laden wind treatment compared to wind alone treatment may reflect an increased need for callus formation and regenerative repair after severe mechanical injury (Mazur et al., 2016). In contrast, the overall decline in Zeatin levels suggests that plants may actively suppress growth processes, such as cell division, while redirecting more resources toward defense and repair. This aligns with the general principle of “inhibiting growth to ensure survival” (Cortleven et al., 2019; Zhang et al., 2020).

Notably, Zeatin and GA levels under sand-laden wind conditions remained higher than those under wind alone treatment, suggesting that, despite overall growth suppression following injury, plants continue to activate limited cell division and elongation programs to initiate localized emergency repair (Lup et al., 2016; Ikeuchi et al., 2019). The concurrent increase in JA, ABA, and SA, alongside the suppression of IAA, GA, and Zeatin, reflects a rapid hormonal reprogramming that prioritizes defense over growth (Nguyen et al., 2016). This early signaling response likely represents the initial phase of the growth–defense trade-off. The synergistic burst of JA, ABA, and SA can induce an excessive defense response to wind-and sand stress, while the contrasting changes in IAA, GA, and Zeatin levels reflect the complex survival strategies adopted by plants when resources are limited. This hormone−mediated “growth-defense” balance indicates that plants utilize different regulatory strategies in response to wind alone and sand-laden wind stress, and may help explain the underlying mechanism by which wind-sand stress induces more severe damage than wind alone. Importantly, this growth−defense trade−off driven by hormonal antagonism is not unique to tomato under wind−sand stress; a recent multi−omics study in Panax ginseng similarly demonstrated that growth retardation results from spatiotemporal antagonism between JA and auxin signaling, redirecting carbon flux from primary growth to secondary metabolism (Yang et al., 2025), suggesting that JA-IAA antagonism may represent a conserved regulatory module through which plants prioritize defense over growth under adverse conditions. Nevertheless, verifying whether this early hormonal reprogramming leads to a sustained growth–defense trade-off will require post-treatment recovery experiments and direct assessment of biomass allocation.

Furthermore, there is another research limitation that needs to be explained: stress intensity in sand-laden wind treatments was defined solely by wind speed, without quantitative measurement of sand flux. Because sand transport flux increases nonlinearly with wind speed, higher velocities likely involve not only greater kinetic energy but also more frequent impacts and increased spatial heterogeneity. This limits the precision of dose–response relationships. Future studies should incorporate quantitative sand flux measurements across different wind speeds and heights to allow a more mechanistic understanding.

## 5 Conclusion

In summary, wind alone stress was observed to affect the physiological functions of tomato seedlings primarily through mechanical stimulation and water stress, while stress induced by sand-laden wind included additional mechanical damage caused by the impact of sand particles, thereby intensifying physiological stress, under severe conditions, potentially leading to seedling death. The mechanical injury induced by wind-sand stress triggers a burst of JA, which acts together with ABA and SA signaling to activate systemic defense, and inhibits the synthesis of growth-related hormones. Such responses likely reflect a restructuring of the regulatory signaling network, directing resources to defense and repair at the expense of growth, indicative of an early hormonal signaling pattern consistent with a growth–defense trade-off. Concurrently, under severe sand-laden wind stress, if the degree of damage exceeds the self-regulatory capacity of the seedlings, this can result in the dysfunction of cell membranes and ROS generation that exceeds the scavenging capacity. This can, in turn, lead to failure of the antioxidant enzyme system and subsequent degradation of proteins as compensation, thereby disrupting normal metabolism. This establishes a vicious cycle that ultimately leads to the collapse of cellular physiological homeostasis. Therefore, severe sand-laden wind stress initiates a cascade that begins with the perception of mechanical signals and ends in comprehensive systemic dysfunction, with the presence of sand particles representing the core factor responsible for this intensification.

The findings of this study provide a theoretical basis for the stress-resistant cultivation of tomatoes and the prevention of field wind damage in regions where wind and sand events are frequent. Future research should focus on the elucidation of plant adaptation strategies under long-term stress, as well as the influence of transported sand, and the physicochemical properties of the sand particles themselves.

## Data Availability

The raw data supporting the conclusions of this article will be made available by the corresponding author, without undue reservation.
