# Supplementary figures and images for: Physiological responses and early hormonal signals associated with growth-defense trade-offs in tomato seedlings under wind-sand stress
Source: Front Plant Sci. 2026 Apr 7;17:1778781. doi: 10.3389/fpls.2026.1778781 (PMC13095534; doi:10.3389/fpls.2026.1778781)

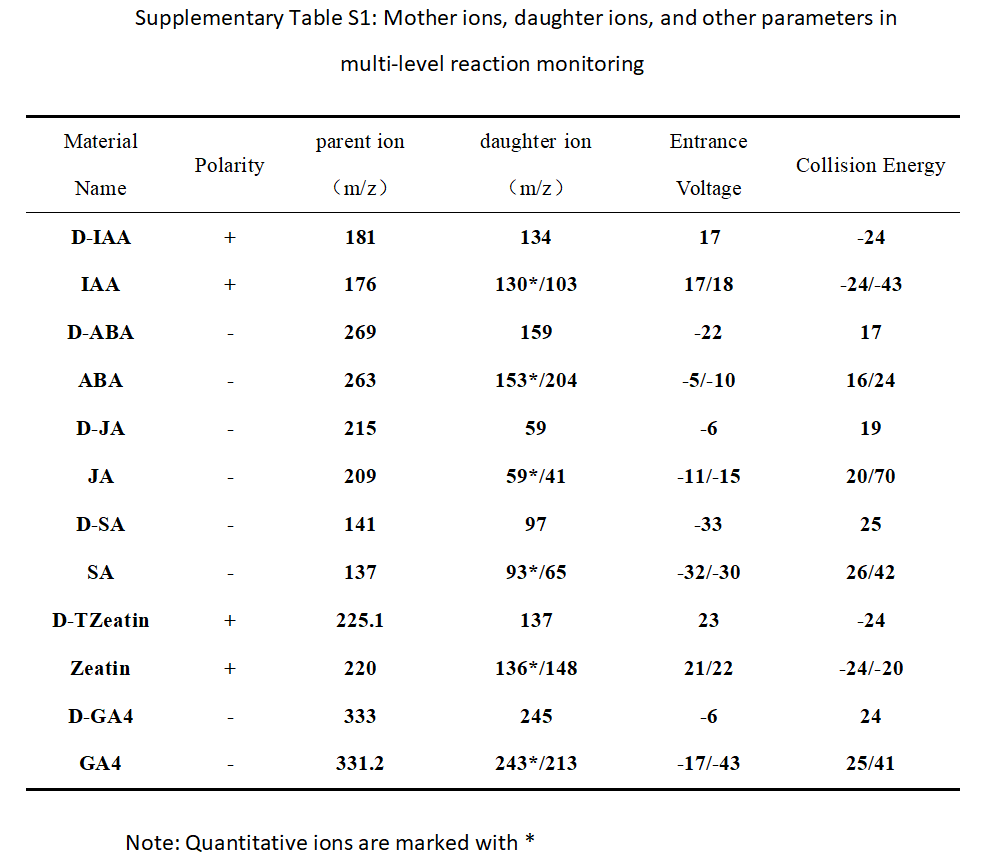

Supplement: Supplementary file 1 [file SupplementaryFile1.tif]
